# Inequalities in Preterm Birth in England: A Retrospective National Cohort Study Focusing on Deprivation and Ethnicity, Using Routinely Collected Maternity Hospital Data

**DOI:** 10.1111/1471-0528.18331

**Published:** 2025-08-22

**Authors:** Iona Hindes, Buthaina Ibrahim, Jennifer Jardine, Dominik Zenner, Stamatina Iliodromiti

**Affiliations:** ^1^ Queen Mary University of London London Greater London UK; ^2^ Royal College of Obstetricians and Gynecologists London Greater London UK

**Keywords:** England, health inequalities, preterm birth

## Abstract

**Objective:**

To quantify the interplay between socioeconomic and ethnic inequalities in preterm birth rates in England from 2018 to 2021.

**Design:**

A retrospective cohort study using electronic health data.

**Setting:**

English hospitals.

**Population:**

1537 595 women aged 13–55 with a singleton livebirth (April 2018–March 2021) at 24–42 gestational weeks were included.

**Methods:**

Multivariate Poisson regression was used to estimate the rate of preterm birth in each ethnic and deprivation group, adjusted rate ratios between groups, and associations. A post hoc calculation identified the rate of preterm birth for each ethnic group at each level of deprivation.

**Main Outcome Measures:**

Preterm birth (birth at less than 37 gestational weeks).

**Results:**

The rate of preterm birth was 6.30% (95% CI: 6.22–6.37) in women living in the most deprived areas, compared to a rate of 5.05% (95% CI: 4.96–5.14) among women in the least deprived areas. White women had a preterm birth rate of 5.74% (95% CI: 5.70–5.78), whereas South Asian and Black women had higher rates of preterm birth at 6.09% (95% CI: 5.98–6.21) and 5.89% (95% CI: 5.70–6.09), respectively. Deprivation interacted with ethnicity and attenuated the differences in the rate of preterm birth across all ethnicity groups (*p* < 0.001). In areas of high deprivation, preterm birth rates were similar across ethnicity groups, whereas in the least deprived areas, South Asian and Black women had higher rates.

**Conclusion:**

Deprivation and ethnicity remain key drivers of inequalities in preterm birth. Prevention strategies need to address socioenvironmental and structural determinants of preterm birth in areas of high deprivation and minority ethnicity groups.

## 1 Introduction

Preterm birth, defined as birth over 24 and under 37 gestational weeks, is an adverse birth outcome associated with severe developmental complications and contributes to neonatal morbidity and mortality globally [1]. There are two types of preterm birth, iatrogenic or spontaneous; the first is when labour is medically induced, the latter is when labour begins preterm without medical intervention. In 2021, after 3 years of sustained decreases, the overall rate of preterm birth in the United Kingdom (UK) increased from 7.4% to 7.5% [2]. This is in keeping with long‐term trends in preterm birth in the UK, in which the rate of preterm birth has remained around 7.5% since 2007 [2]. Across North America, Australia, New Zealand, central Asia and Europe, the rate of preterm birth has also remained consistent, between 7.8% and 7.9%, over the last decade [3]. In the United States, preterm birth is particularly high at over 10% among livebirths; this rate also increased in 2021 to 10.5% from 10.1% in 2020 [3, 4].

Preterm birth disproportionately affects women of minority ethnicity and those living in deprivation [5]. The Office of National Statistics (ONS) reported in 2021, women of Black Caribbean ethnicity had the highest rate of preterm birth at 10.2% and between 2020 and 2021 preterm birth rates increased the most among Asian women, from 7.5% to 8.1% [2]. Previous research estimated 18.5% of preterm births ‘could be attributed to socioeconomic inequality’ [5]. Recent evidence showed that overall preterm birth decreased in high‐income countries during lockdown restrictions implemented for the coronavirus disease of 2019 (COVID‐19) pandemic, with women from White backgrounds experiencing the highest decrease compared to other ethnicity groups [6, 7, 8]. However, inequalities in preterm birth have persisted during and after lockdown restrictions, and regardless of increased awareness and initiatives to reduce them [1, 3, 8]. The unequal burden of preterm birth perpetuates health disadvantage in ethnic minority and deprived communities [1]. To sustainably reduce inequalities in preterm birth, better evidence is needed to develop public health policies and interventions which enable equity [1, 3, 5].

### 1.1 Statement of Study Objective

In this paper we aim to quantify socioeconomic and ethnic inequalities in preterm birth, and explore the interaction between ethnicity and deprivation, using routinely collected maternity hospital admissions data in England from 2018 to 2021 when a period of sustained decreases in preterm birth ended [6, 7].

## 2 Methods

This is a retrospective cohort study which used admitted patient care (APC) dataset from the English National Health Service (NHS), Hospital Episode Statistics (HES) database. HES is a national database covering all hospital care paid for by the NHS in England. It includes data on all inpatient hospital admissions and episodes, anonymised and accessed for research purposes [9]. It is highly representative of the hospitalised population and includes most births (approximately 97%) in England [10, 11].

For this study we obtained access to anonymized admission patient care data for all women who had a recorded birth from 1st April 2018 to 31st March 2021, and historical data on all hospital admissions for these patients going back to 1st January 2000. Data available includes self‐reported ethnicity, age, and Index of Multiple Deprivation (IMD), number of babies born, gestational age at birth, delivery method, current and historical diagnosis codes, operation and procedure (OPCS‐4) codes. The 10th revision of the International Classification of Diseases, known as ICD‐10, and the 4th version of Office for Population Censuses and Surveys Classifications of Interventions and Procedures, known as OPCS‐4, were used to identify maternity records, outcomes, parity, previous adverse obstetric outcomes, maternal comorbidities, and COVID‐19 infection; codes are described in the Supporting Information (Table S1). Variables regarding maternal health (including previous episodes, deliveries, and diagnoses) and demographic information (such as IMD or ethnicity) are recorded at the time of birth or from previous birth admission records.

Data was held and managed by the Royal College of Obstetricians and Gynaecologists (RCOG) and accessed through a remote access gateway. The NHS Health Research Authority recognises this project as a service evaluation which did not require further ethical approval. Approvals for the use of de‐identified HES data were obtained as part of the standard NHS Digital approval process.

**FIGURE 1:**
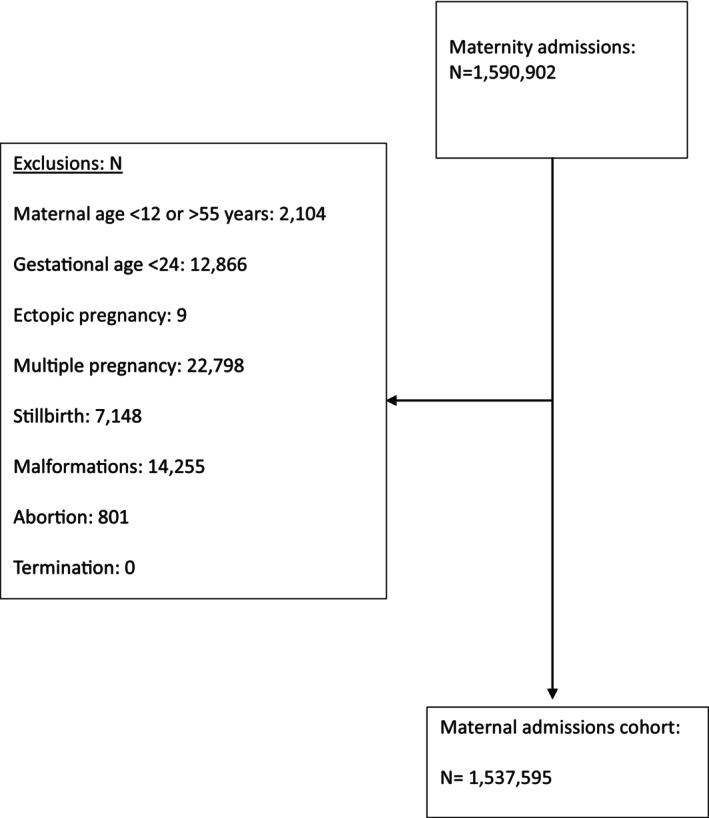
Flowchart outlining cohort selection and the number of excluded records.

All women (this study refers to patients as women for ease of readership but acknowledges that not all individuals who give birth are/identify as women and uses this term as inclusively as possible) aged between 13 and 55 years who had a singleton livebirth between 1st April 2018 and 31st March 2021 were included. Multiple pregnancies were excluded from this study as drivers of preterm birth in multiple pregnancies are different from those in singletons and could confound associations. Births were included if their gestation was over 24 and under 42 completed weeks. Pregnancies recorded as ectopic, terminated, or aborted, and any admissions identified as a miscarriage, malformation of foetus, or stillbirth, were excluded. The derivation of the cohort is described in Figure 1.

The primary outcome was preterm birth defined as birth of an infant between 24 and up to 37 gestational weeks. In supplementary analysis, secondary outcomes relate to the mother and include the two types of preterm birth, which are medically indicated (iatrogenic) and spontaneous preterm birth. Medically indicated preterm birth, also known as iatrogenic preterm birth, is when a mother undergoes a planned preterm delivery because of medical indications. Conversely, spontaneous preterm birth is where the mother's labour starts without medical intervention.

Socioeconomic deprivation was determined using the address registered with their hospital at the time of birth and derived from the IMD 2019 estimates [12]. IMD measures relative levels of neighbourhood deprivation or small areas of approximately 1000–3000 people, known as Lower‐layer Super Output Areas (LSOAs) in England [12, 13]. It is a composite index of numerous indicators which are divided into several domains. Domains range from individual income, education, and employment to environment such as living environment, crime, barriers to housing, and services. These domains are allocated weights and combined to give an overall estimate of the level of relative deprivation in a neighbourhood. Each area is allocated to a quintile; the 1st quintile includes the 20% least deprived areas and the 5th quintile the 80%–100% most deprived. Ethnicity was obtained through previous admission records; it was self‐reported and subsequently classified based on the ONS Census ethnicity categories. Census‐based categories were collapsed into 5 aggregated ethnicity groups: White, Mixed, South Asian, Black, and Other (Table S2) [14]. Ethnicity in HES has a good overall agreement rate (over 89%) at the aggregated ethnicity level with ethnicity reported by individuals in the UK Census [15]. Supporting Information (Table S3) describes other variables that were used in our models: maternal age, year of delivery, maternal comorbidities, parity, previous adverse birth and pregnancy outcomes, COVID‐19 infection, and lockdowns (Table S3) [7, 16, 17, 18, 19]. Lockdowns were defined by the government timelines of national and regional lockdown restrictions [20]. Lockdowns were adjusted for due to their association with reduced rates of preterm birth.

The frequency of premature birth, characteristics of women in the cohort, and frequency of missing data were explored in descriptive analysis. Missing data was addressed using multiple imputation of missing values for model covariates (deprivation, ethnicity, year, and gestational age). We imputed missing data by using chained equations to generate 10 imputed datasets, which were combined using Rubin's rules [21].

We used multivariate Poisson regression, with robust standard errors to account for clustering within the sample, to estimate incidence risk ratios of preterm birth at each level of deprivation. Clusters can occur when individuals have had several births; robust standard errors widen the confidence intervals within these clusters to enable comparison between individuals [22]. In the bivariate analysis, we adjusted for ethnicity and deprivation to estimate the rate of preterm birth in each ethnicity and deprivation group, and the relative rate compared to White women living in the 20% least deprived areas. We iteratively developed the model, adjusting for confounders or risk factors associated with preterm birth and informed by previous research [22]. A *p*‐value of less than 0.05 was considered statistically significant. The final model adjusted for all risk factors, confounders, and interactions. An interaction term between ethnicity and deprivation was generated to capture the effects between these variables and was adjusted for in the final model [5, 23]. Interaction terms were included where they improved the model fit. Model fit was compared using likelihood ratio tests (LRT), Wald tests, and Akaike's Information Criterion (AIC); if the addition of the interaction term in the model resulted in LRT and Wald tests with a *p*‐value less than 0.05 and a smaller AIC, it was adjusted for in the model. A post hoc calculation was run to identify the rate of premature birth for each ethnicity group at each level of deprivation. These estimates were then formatted into a two‐by‐two table and conditionally formatted to display a ‘heatmap’ in which higher rate estimates had a darker colour orange. Supplementary analysis explored inequalities according to deprivation and ethnicity in distinct classifications of medically indicated and spontaneous preterm birth separately.

Two sensitivity analyses were conducted. First, we tested whether our results were affected by COVID‐19 by excluding women with confirmed COVID‐19 infection from the analysis and comparing this to our main results. COVID‐19 infection diagnosis codes were of laboratory‐confirmed COVID‐19 viral infection. Second, we explored differences in preterm birth estimates between the imputed and complete‐case datasets. The amount of missing data for each variable was calculated; any observation missing data on the outcome, exposure, or covariate of interest was excluded from the complete case subset. A complete case subset included women with complete data across all variables (70% of livebirths). The regression was repeated on the complete case subset, and estimates were compared with imputed results [21].

All analysis was conducted in Stata 16.1 (StataCorp, College Station, TX).

This study was funded by the National Institute of Health Research: School for Primary Care Research, the Healthcare Improvement Studies Institute, the Health Foundation, and Tommy's National Preterm Birth Research Centre. The views expressed are those of the author(s) and not necessarily those of the funders.

Patients were not involved in the development of this research.

## 3 Results

We identified 1 590 902 women with maternity admissions between April 2018 and March 2021, of whom 1 537 595 had livebirths and were eligible for inclusion (Figure 1). Descriptive details can be found in Table 1.

**TABLE 1:** Descriptive characteristics of all included livebirths in the dataset (imputed dataset).

Variables		Frequency (*N*)	Percent (%)	Frequency of observations missing data (%)
Total livebirths		1 537 595	100	0
Preterm birth	Preterm births	97 572	6.35	0
Gestational age at birth (weeks)	< 37	76 070	6.33	335 274 (21.81%)
	≥ 37 & < 42	1 102 631	91.7	
	42 ≥	23 620	1.96	
Classification of preterm labour and birth (medically indicated or spontaneous)	Iatrogenic	37 095	2.41	0
	Spontaneous	44 587	2.90	
Socio‐demographic characteristics				
Socioeconomic deprivation	Least deprived 20%	227 642	15.5	63 768 (4.15%)
	Less deprived 20%–30%	244 485	16.6	
	Median deprived 40%–50%	322 787	21.9	
	More deprived 60%–80%	325 450	22.1	
	Most deprived 80%–100%	353 463	24.0	
Ethnicity group	White	1 139 833	76.6	48 978 (3.19%)
	Mixed	31 196	2.10	
	South Asian	175 463	11.8	
	Black	72 215	4.85	
	Other	69 910	4.70	
Maternal age	13–19	41 904	2.73	13 (0%)
	20–34	1 133 761	73.7	
	35–44	357 100	23.2	
	45–55	4817	0.31	
Admissions per year	2018	393 172	25.6	2401 (0.16%)
	2019	496 871	32.4	
	2020	521 606	34.0	
	2021	123 545	8.05	

Overall, there were 97 572 preterm births (6.35% of all births) in the cohort. The rate of preterm birth was highest in women living in the most deprived 20% of areas (7.05%), and in women of South Asian (7.13%) and Black (7.25%) ethnicity (Table S4). The lowest rates of preterm birth were among women living in the least deprived 20% of areas (5.14%), women of White (6.24%) and Other (5.70%) ethnicity groups (Table S4).

In the fully adjusted model, when all risk factors, confounders, and the interaction between ethnicity and deprivation were included, the rate of preterm birth was lowest in women living in the 20% least deprived areas at 5.05% (95% CI: 4.96–5.14) (Table 2). The rate of preterm birth was 1.29 (95% CI: 1.26–1.33) times higher among women living in the 80%–100% most deprived areas. White women had a preterm birth rate of 5.74% (95% CI: 5.69–5.78). South Asian women had higher rates of preterm birth at 6.09% (95% CI: 5.97–6.20); so did Black women at 5.89% (95% CI: 5.69–6.08). The rate of preterm birth was 1.16 (95% CI: 1.08–1.24) times higher among South Asian women, and 1.15 (95% CI: 1.01–1.31) times higher among Black women, compared to White women (Table 2).

**TABLE 2:** Preterm birth rates in each area of deprivation, risk ratios comparing subgroups to the reference, in the multiple imputed datasets (Unadjusted bivariate analysis is the crude rates are presented first followed by multivariate model fully adjusted for risk factors).

Total livebirths: 1537595		Model 1: unadjusted model, bivariate analysis			Model 2: fully adjusted model^a^, multivariate analysis		
		Constant baseline rate per 100 livebirths (95% CI): 6.22 (6.17–6.26)			Constant baseline rate per 100 livebirths (95% CI): 5.34 (5.23–5.47)		
		Estimates			Estimates		
Characteristics	Subgroup of variable	Rate per 100 livebirths (95% CI)	Risk ratio (95% CI)	*p*	Rate per 100 livebirths (95% CI)	Risk ratio (95% CI)	*p*
Deprivation	Least deprived 20%	5.24 (5.15–5.33)	Reference (Ref)	< 0.001	5.05 (4.96–5.14)	Ref	< 0.001
	Less deprived 20%–40%	5.59 (5.50–5.68)	1.07 (1.04–1.09)		5.28 (5.19–5.37)	1.05 (1.03–1.08)	
	Median deprived 40%–60%	6.67 (6.59–6.76)	1.27 (1.25–1.30)		5.97 (5.90–6.05)	1.20 (1.18–1.23)	
	More deprived 60%–80%	6.58 (6.50–6.67)	1.26 (1.23–1.28)		5.90 (5.82–5.98)	1.19 (1.16–1.22)	
	Most deprived 80%–100%	7.07 (6.99–7.15)	1.35 (1.32–1.38)		6.29 (6.22–6.37)	1.29 (1.26–1.33)	
Ethnicity	White	6.22 (6.17–6.26)	Ref	< 0.001	5.74 (5.69–5.78)	Ref	< 0.001
	Mixed	6.48 (6.21–6.75)	1.04 (1.00–1.09)		5.74 (5.50–6.00)	1.03 (0.90–1.19)	
	South Asian	7.08 (6.96–7.20)	1.14 (1.12–1.16)		6.09 (5.97–6.20)	1.16 (1.08–1.24)	
	Black	7.20 (7.01–7.39)	1.16 (1.13–1.19)		5.89 (5.69–6.08)	1.15 (1.01–1.31)	
	Other	5.70 (5.53–5.87)	0.92 (0.89–0.95)		5.21 (5.04–5.38)	0.94 (0.84–1.05)	

^a^ Adjusted for ethnicity, an interaction term between deprivation and ethnicity, age, year, parity, and previous adverse birth outcomes, maternal comorbidities, COVID‐19 infection, and lockdowns.

An interaction term between deprivation and ethnicity significantly contributed to the model fit (*p* < 0.001) and justified further stratified analysis, in which we compared the rate of preterm birth in each ethnicity group at each quintile of area deprivation. The heatmap in Table 3 shows the rate of premature birth increased according to deprivation across all ethnicity groups. Among women living in the least deprived area, the rate of premature birth was lowest in White and Other ethnicity women (4.9%, 95% CI: 4.8%–5.0% & 4.6%, 95% CI: 4.2–5.2) and highest in South Asian and Black women (5.7%, 95% CI: 5.4%–6.1% & 5.7%, 95% CI: 5.0–6.5) (Table 3). In the most deprived quintile, the rate of preterm birth was similar across ethnicity groups (Table 3).

**TABLE 3:** Rate of premature birth per 100 livebirths (95% CI), in each ethnicity group per deprivation quintile based on estimates from the multivariate model.

	White	Mixed	South Asian	Black	Other
Least deprived 20%	4.9% (4.8–5.0)	5.1% (4.4–5.9)	5.7% (5.4–6.1)	5.7% (5.0–6.5)	4.6% (4.2–5.2)
Less deprived 20%–40%	5.2% (5.1–5.3)	5.7% (5.0–6.4)	5.8% (5.5–6.1)	5.6% (5.0–6.2)	4.8% (4.3–5.3)
Median deprived 40%–60%	6.0% (5.9–6.0)	5.7% (5.2–6.2)	6.3% (6.1–6.5)	6.2% (5.9–6.5)	5.5% (5.2–5.8)
More deprived 60%–80%	5.9% (5.8–6.0)	5.8% (5.3–6.3)	6.3% (6.1–6.5)	5.9% (5.6–6.3)	5.3% (5.0–5.7)
Most deprived 80%–100%	6.4% (6.3–6.5)	6.2% (5.8–6.7)	6.1% (6.0–6.3)	6.0% (5.7–6.2)	5.6% (5.3–5.9)

Subgroups with higher preterm birth rate estimates, are marked by a darker colour orange as the fill colour.

Supplementary analysis found the association was consistent in spontaneous and medically indicated preterm birth; the rate of spontaneous and medically indicated preterm birth increased according to increasing area level deprivation (Tables S7 and S8). The effect of ethnicity on medically indicated preterm birth was also consistent with the overall preterm birth (Table S8). The association of ethnicity with spontaneous preterm birth differed considerably when all covariates were adjusted for. White women had the highest rate of spontaneous preterm birth at 2.92% (95% CI: 2.88–2.95), whereas South Asian and Black women had a lower spontaneous preterm birth rate of 2.74% (95% CI: 2.66–2.83) and 2.51% (95% CI: 2.38–2.64), respectively. Rates were 0.94 times lower among South Asian women (95% CI: 0.91–0.97) and 0.86 times lower among Black women (95% CI: 0.81–0.91), compared to White women (Table S7).

When individuals who tested positive for COVID‐19 in pregnancy were removed from the sample, the observed patterns of association between preterm birth, deprivation and ethnicity remained consistent (Table S9).

Our complete case sample included 1 111 045 women (70% of total sample), who had no missing data in any of the variables (Table S5). Results from imputed datasets were consistent with complete case estimates (Tables S5 and S6). The estimates for Black women did not reach statistical significance in the complete case subset, in which the rate of preterm among Black women did not differ significantly from the rate among White women (1.09, 95% CI: 0.94–1.27) (Tables S5 and S6).

## 4 Discussion

### 4.1 Main Findings

In this study of over one million births in England from 2018 to 2021, we found persistent evidence of considerable inequalities in the rate of preterm birth between areas of deprivation and ethnicity groups. We also found that area deprivation and ethnicity interacted to impact the rate of preterm birth. Women living in the most deprived areas had up to a 30% increase in the rate of preterm birth compared to women living in the least deprived areas. In the least deprived areas, the rate of preterm birth was 15%–16% higher among South Asian and Black women than among White women.

### 4.2 Strengths and Limitations

Our research included almost all births (approximately 97%) in England between 2018 and 2021 [9]. Multivariate analysis was developed iteratively and adjusted for key risk factors of preterm delivery. However, this dataset lacked information on key risk factors of smoking status and body mass index (BMI) [16]. Whilst we have used proxies, such as obesity, to mitigate the impact of high BMI, we acknowledge that obesity may be underreported in administrative healthcare databases and classifications may vary in accuracy between ethnicity groups [24]. Furthermore, area‐level deprivation was used rather than individual deprivation estimates, which limits the ability to identify which socioeconomic factors directly or indirectly influence preterm birth rates [25]. To mitigate this limitation, we included individual ethnicity and an interaction term between ethnicity and deprivation to explore individual socioeconomic factors in more detail. A further limitation is the possibility of inaccuracy in digital records. This is a limitation of all research in electronic health records and it is not practical to directly audit records. However, postcode at delivery is used in England for in‐home postnatal care, and diagnosis codes are used for hospital payment purposes, suggesting that these codes are likely to be largely accurate.

Despite the relatively high completeness (70% complete data) of our data, especially for deprivation (4.15% missing) and ethnicity (3.19% missing), the rate of data missing on deprivation was higher among non‐White women (Mixed: 4.71%, South Asian: 4.88%, and Other: 5.54%) compared to White women (3.83%). Black women had the most data missing on deprivation (6.29%). Sensitivity analysis found that the complete case dataset was underpowered to investigate the effect of ethnicity and deprivation on preterm birth. To mitigate this, we used robust methodology to impute data, thereby increasing the power of our sample to more accurately assess the effect of deprivation and ethnicity on preterm birth.

In this dataset, there are further concerns regarding the accuracy of the recorded ethnicity data. The ONS has reported lower levels of agreement with ethnicity coding among ethnic minorities, with the lowest levels of agreement (67% and 69%) among Mixed and Other ethnicity groups [15]. Therefore, the effect of ethnicity could be underestimated in this sample due to inaccuracy in ethnicity coding. Furthermore, most births in England take place in NHS hospitals; however, some do not, for instance, immigrants without legal documentation, those who give birth without the support of clinicians, or those in private hospitals. These patients represent the most vulnerable and most affluent members of society; thus, the effect of deprivation could be underestimated.

## 5 Interpretation

The stratified rates of preterm birth in ‘heatmaps’ indicate that across all ethnicity groups, deprivation increases the rate of prematurity by up to 31%. In the most deprived areas, the rate of preterm birth was similar across ethnic groups. This association was consistent for iatrogenic and spontaneous preterm births. Previous research in England has identified social deprivation as a risk factor for preterm birth [5, 22]. Our findings are consistent with previous research and indicate that despite concerted efforts to reduce inequalities in recent years and the decreased rates of preterm birth observed during periods of lockdown restrictions, deprivation remains an important determinant of preterm birth [5, 8].

The higher rate of preterm birth in deprived settings may be partially explained by a multitude of factors, such as a higher prevalence of environmental and behavioural risk factors, including smoking and higher BMI [16]. Health inequalities are widening in the UK due to cuts in central government's and local authorities' spending power [26]. This decrease in funding to support local initiatives, which improve health and living standards in areas of high deprivation, leads to greater health disparities [26]. Therefore, targeted action and investment to support healthy lifestyles and access to care are required to sustainably reduce preterm birth.

Unlike previous research, we identified a significant interaction between ethnicity and deprivation, in which ethnicity attenuates the rate of preterm births across deprivation areas. Stratified analysis found that in the least and low deprivation areas, South Asian and Black women remained at a disproportionately increased rate compared to White women. The highest rates of preterm birth (6.2%–6.4%), seen in the most deprived areas among White and Other women, are observed in lower levels of deprivation (median 40%–50% deprived areas and 60%–80% more deprived areas) among South Asian and Black women.

Previous research has suggested that higher rates of maternal comorbidities, multiple morbidities, or higher severity of conditions could be responsible for these inequalities [22]. This is consistent with our results, as our sensitivity analysis found the association between ethnicity and iatrogenic preterm birth largely disappeared when maternal comorbidities were adjusted for. However, higher rates of iatrogenic preterm birth remained among South Asian women. In preterm birth overall, where missing data was imputed and such comorbidities adjusted for, inequalities in rates between ethnicity groups persisted. This indicates a possibility of residual confounding by variables not considered in our analysis due to a lack of data availability; these factors may include unconscious or conscious bias among practitioners, structural racism, or increased stress due to discrimination [27, 28, 29, 30].

As surrounding research has previously suggested, prevention efforts must engage multiple sectors to ensure timely access to healthcare and provide targeted support to those at high risk [5, 22]. Ours and previous analyses indicate that maternal comorbidities contribute to inequalities in iatrogenic preterm birth between ethnicity groups. Primary and secondary prevention should target those living in high areas of deprivation, South Asian and Black women, to reduce the prevalence of comorbidities in these high‐risk groups. Finally, the determinants of both high iatrogenic and spontaneous preterm birth rates among areas of high deprivation are unclear. Further research is required to understand which mechanisms are responsible for high preterm birth rates in deprived areas so effective prevention measures can be developed.

## 6 Conclusion

Inequalities in preterm birth based on socioeconomic status and ethnicity have persisted; it appears these factors interact to unequally burden South Asian and Black women, and women living in high deprivation. The unequal burden of preterm birth perpetuates health inequalities and cycles of intergenerational health inequity [1]. Healthcare services, policy makers, and local authorities need to collaborate to develop interventions tailored to address the needs of South Asian and Black women, and those living in high deprivation areas.

To sustainably reduce inequalities, tailored strategies need to be developed that target vulnerable groups, such as South Asian and Black women [5, 29, 31]. Examples of potential strategies include:

- Updating clinical guidance to identify high‐risk groups and allocate additional maternal and foetal monitoring as required.
- Prioritise the provision of continuity and personalised care to high‐risk patients.

All tailored strategies should be co‐produced with both patients' and practitioners.

## Author Contributions

S.I., D.Z., and I.H. developed the research question and study design. I.H. did the statistical analysis, with input and guidance from S.I., D.Z., B.I., and J.J. B.I. cleaned, prepared, and organised the data and managed access to the data. B.I. and J.J. provided input on data organisation and statistical analysis. I.H. wrote the abstract and manuscript, with input and guidance from D.Z., S.I., B.I., and J.J., D.Z. and S.I. jointly supervised the work. All authors have seen and approved the final version of the abstract and manuscript for publication.

## Conflicts of Interest

J.J. was a co‐author of the RCOG guidelines which determined changes to maternity care during the COVID‐19 pandemic.

## Supporting information


**Data S1:** bjo18331‐sup‐0001‐DataS1.docx.

## Data Availability

Data used in this study included de‐identified patient records, this data is accessible upon request and application through NHS England. Related study documents are available upon request.
