# Supplementary material for: Inequalities in Preterm Birth in England: A Retrospective National Cohort Study Focusing on Deprivation and Ethnicity, Using Routinely Collected Maternity Hospital Data
Source: BJOG. 2025 Aug 22;132(12):1866–74. doi: 10.1111/1471-0528.18331 (PMC12501656; doi:10.1111/1471-0528.18331)
Supplement: Supplementary file 1 — Data S1: bjo18331‐sup‐0001‐DataS1.docx. [file BJO-132-1866-s001.docx]

**Supplementary material**

Table S1. OPCS and ICD-10 diagnosis codes used to identify outcomes, parity, previous adverse birth outcomes, and maternal health covariates.

Table S2. ONS classifications allocated to each ethnicity group.

Table S3. Covariate subgroups and definitions

Table S4. Descriptive table of the rates of preterm birth in imputed dataset, and rates of preterm birth per deprivation and ethnicity group.

Table S5. Rates and rate ratios of preterm birth in complete case dataset.

Table S6. Rate of premature birth per 100 livebirths (95%CI), in each ethnicity group per deprivation quintile, in complete case dataset.

Table S7. Spontaneous premature birth rate and rate ratios in complete case dataset.

Table S8. Iatrogenic premature birth rates and rate ratios in complete case dataset.

Table S9. Premature birth rates and rate ratios in cohort with COVID-19 positive patients excluded, in complete case dataset.

Table S1. Office for Population Censuses and Surveys Classifications of Interventions and Procedures (OPCS), International classification of diseases (ICD-10), and Hospital Episode Statistics (HES) codes used to identify outcomes, parity, previous adverse birth outcomes, and maternal health covariates.

| **Variable** | **Code Type** | **Code Term** | **Description in Relevant Code Book Dictionary** |
| --- | --- | --- | --- |
| **Preterm birth** | ICD10 | P072 | Extreme immaturity |
|  |  | O60 | Preterm labour and delivery |
|  |  | P590 | Neonatal jaundice associated with preterm delivery |
|  | HES | gestat<37 | Length of gestation |
| **Medically indicated Preterm birth** | ICD10 | O603 | Preterm delivery without spontaneous labour (by caesarean section, without spontaneous labour/by induction of labour) |
|  | HES | gestat<37 | Length of gestation |
| **Spontaneous Preterm birth** | ICD10 | 0601 | Preterm spontaneous labour with preterm delivery |
|  | HES | gestat<37 | Length of gestation |
| **Maternal parity and previous adverse birth outcomes** | | | |
| **Parity (previous pregnancy)** | ICD10 | Z37 | Outcome of delivery |
|  | OPCS | R17 – R25 | Elective caesarean delivery  Other caesarean delivery  Breech extraction delivery  Other breech delivery  Forceps cephalic delivery  Vacuum delivery  Cephalic vaginal delivery with abnormal presentation of head at delivery without instrument  Normal delivery  Other methods of delivery |
|  | HES | DELMETH_1 (0-9) | Method used to deliver a baby that is a registerable birth, first baby delivered. |
| **Previous cesarean section** | ICD10 | O82 (0-9) | Single delivery by cesarean section |
|  |  | O842 | Multiple delivery, all by cesarean section |
|  | OPCS | R17 (0-9) | Elective cesarean delivery |
|  |  | R18 (0-9) | Other cesarean delivery |
| **Previous preterm birth** | ICD10 | P072 | Extreme immaturity |
|  |  | O60 | Preterm labour and delivery |
|  |  | P590 | Neonatal jaundice associated with preterm delivery |
|  | HES | gestat<37 | Length of gestation |
| **Previous stillbirth** | ICD10 | P95 | Fetal death of unspecified cause |
|  |  | Z371 | Single stillbirth |
|  |  | Z373 | Twins, one liveborn and one stillborn |
|  |  | Z374 | Twins, both stillborn |
|  |  | Z376 | Other multiple birth, some liveborn |
|  |  | Z377 | Other multiple birth, all stillborn |
|  | HES | birstat=2-4 | Birth status (stillbirth ante-partum, intra-partum and indeterminate) |
|  |  | dismeth=5 | Method of discharge (baby was still born) |
| **Maternal comorbidities** | | | |
| **Maternal hypertensive conditions and pre-eclampsia** | ICD10 | O10 | Pre-existing hypertension complicating pregnancy, childbirth, and puerperium. |
|  |  | O11 | Pre-eclampsia superimposed on chronic hypertension |
|  |  | O13 | Gestational hypertension (pregnancy induced hypertension) |
|  |  | O14 | Pre-eclampsia |
|  |  | O16 | Unspecified maternal hypertension |
|  |  | I10 | Essential (primary) hypertension |
|  |  | O150 | Eclampsia in pregnancy |
|  |  | O151 | Eclampsia in labour |
| **Diabetes** | ICD10 | O24 | Diabetes mellitus in pregnancy |
|  |  | E10 | Type 1 diabetes mellitus |
|  |  | E11 | Type 2 diabetes mellitus |
| **Obesity** | ICD10 | E66 | Obesity |
| **COVID-19 Infection** | | | |
| **COVID-19 infection** | ICD10 | U07.1 | Confirmed laboratory testing of COVID-19 virus identified |

Table S2. Office for National Statistics (ONS) classifications allocated to each ethnicity group.

| Ethnicity | HES Ethnos variable, ethnic categories based on 2001 Census |
| --- | --- |
| White | A = British (White)  B = Irish (White)  C = Any other White background |
| Mixed | D = White and Black Caribbean (Mixed)  E = White and Black African (Mixed)  F = White and Asian (Mixed)  G = Any other Mixed background |
| Asian | H = Indian (Asian or Asian British)  J = Pakistani (Asian or Asian British)  K = Bangladeshi (Asian or Asian British)  L = Any other Asian background |
| Black | M = Caribbean (Black or Black British)  N = African (Black or Black British)  P = Any other Black background |
| Other | R = Chinese (other ethnic group)  S = Any other ethnic group |

Table S3. Covariate subgroups and definitions

*at least one previous case or diagnosis

| Variable | Subgroups |
| --- | --- |
| Age at admission | 13 – 19,  20-34 (reference group),  35-44,  and 45-55 |
| Maternal comorbidities | No comorbidities,  Diagnosis of at least one comorbidity (hypertensive conditions, pre-eclampsia, diabetes, obesity) |
| Parity and previous cesarean section * | Primiparous (first time mother),  Multiparous and all previous vaginal births,  Multiparous and previous cesarean section |
| Parity and previous preterm birth * | Primiparous (first time mother),  Multiparous and all previous full term births,  Multiparous and previous preterm birth |
| Parity and previous stillbirth * | Primiparous (first time mother),  Multiparous and all previous livebirths,  Multiparous and previous stillbirth |
| COVID-19 infection * | No positive covid test in pregnancy,  Positive covid test in pregnancy or birth |
| Lockdown restrictions | Pre-lockdown (1^st^ April 2018 – 23^rd^ March 2020),  Lockdown 2020 (24 March – 23 June 2020),  Tier restrictions (24^th^ June – 5^th^ November 2020), and  Lockdown 2020-2021 (5^th^ November – 31^st^ March 2021)). |

Table S4. Descriptive table of the rates of preterm birth in imputed dataset, and rates of preterm birth per deprivation and ethnicity group.

|  | **Total live births** | **Frequency of preterm births (N) & rate of preterm birth (%)** |
| --- | --- | --- |
| **Total Cohort** | 1,537,595 | 97,572 (6.35) |
| **Deprivation Quintile** | 1,473,827 (total livebirths with information on deprivation quintiles) | |
| Least deprived 20% | 227,642 | 11,693 (5.14) |
| Less deprived 20-30% | 244,485 | 13,457 (5.50) |
| Median deprived 40-50% | 322,787 | 21,408 (6.63) |
| More deprived 60-80% | 325,450 | 21,286 (6.54) |
| Most deprived 80-100% | 353,463 | 24,907 (7.05) |
| **Ethnicity** | 1,488,617 (total livebirths with information on ethnicity group) | |
| White | 1,139,833 | 71,164 (6.24) |
| Mixed | 31,196 | 2,033 (6.52) |
| South Asian | 175,463 | 12,510 (7.13) |
| Black | 72,215 | 5,234 (7.25) |
| Other | 69,910 | 3,985 (5.70) |

Table S5. Rates, and rate ratios of preterm birth in complete case dataset (Multivariate models adjusted for risk factors as follows:

**Final Model:** Adjusted for deprivation, ethnicity, an interaction term between deprivation and ethnicity, age, year, parity and previous adverse birth outcomes, maternal comorbidities, COVID-19 infection, and lockdowns)

| **Variables** | | **Final Model** | | |
| --- | --- | --- | --- | --- |
| **Risk factor variable** | **Subgroup of variable** | **Rate per 100 livebirths (95%CI)** | **Rate ratio (95%CI)** | **P value** |
| Deprivation | Least deprived 20% | 5.62 (5.50-5.74) | Ref | <0.001 |
|  | Less deprived 20-40% | 6.00 (5.88-6.12) | 1.07 (1.04-1.10) |  |
|  | Median deprived 40-60% | 6.78 (6.68-6.88) | 1.22 (1.19-1.26) |  |
|  | More deprived 60-80% | 6.75 (6.65-6.85) | 1.22 (1.18-1.25) |  |
|  | Most deprived 80-100% | 7.10 (7.00-7.20) | 1.30 (1.27-1.34) |  |
| Ethnicity | White | 6.52 (6.47-6.58) | ref | 0.0117 |
|  | Mixed | 6.67 (6.35-6.99) | 1.05 (0.90-1.23) |  |
|  | South Asian | 6.90 (6.76-7.04) | 1.13 (1.05-1.22) |  |
|  | Black | 6.63 (6.39-6.88) | 1.09 (0.94-1.27) |  |
|  | Other | 5.94 (5.73-6.16) | 0.94 (0.83-1.06) |  |
| Age | 13-19 | 8.78 (8.46-9.11) | 1.38 (1.33-1.43) | <0.001 |
|  | 20-34 | 6.38 (6.33-6.43) | Ref |  |
|  | 35-44 | 6.73 (6.63-6.83) | 1.06 (1.04-1.07) |  |
|  | 45-55 | 10.23 (9.08-11.37) | 1.60 (1.43-1.79) |  |
| Year | 2018 | 7.37 (7.25-7.48) | ref | <0.001 |
|  | 2019 | 6.28 (6.18-6.38) | 0.85 (0.84-0.87) |  |
|  | 2020 | 6.20 (6.08-6.32) | 0.84 (0.82-0.87) |  |
|  | 2021 | 5.98 (5.71-6.24) | 0.81 (0.77-0.85) |  |
| Parity and previous cesarean section (CS) | Primiparous | 3.57 (3.51-3.62) | ref | <0.001 |
|  | Multiparous & vaginal delivery | 30.13 (27.05-33.22) | 8.45 (7.54-9.48) |  |
|  | Multiparous & previous cs | 37.81 (33.99-41.63) | 10.6 (9.47-11.9) |  |
| Parity and previous preterm birth (PTB) | Primiparous | 15.25 (3.55-26.70) | Ref | <0.001 |
|  | Multiparous & fullterm | 3.11 (2.49-3.73) | 0.20 (0.08-0.53) |  |
|  | Multiparous & previous PTB | 12.71 (10.17-15.24) | 0.83 (0.32-2.16) |  |
| parity and previous stillbirth (SB) | Primiparous | 11.39 (3.55-19.22) | Ref | <0.001 |
|  | Multiparous & livebirths | 4.62 (3.38-5.86) | 0.41 (0.16-1.06) |  |
|  | Multiparous & previous SB | 5.56 (4.04-7.09) | 0.49 (0.19-1.27) |  |
| Maternal comorbidities | None | 5.81 (5.76-5.87) | ref | <0.001 |
|  | Conditions diagnosed | 8.23 (8.13-8.32) | 1.42 (1.39-1.44) |  |
| COVID-19 | Negative covid test | 6.51 (6.47-6.56) | ref | <0.001 |
|  | Positive covid test | 11.7 (10.8-12.6) | 1.80 (1.66-1.94) |  |
| Lockdown period | Pre-lockdown | 6.63 (6.55-6.71) | ref | 0.0068 |
|  | Lockdown 2020 | 6.39 (6.19-6.59) | 0.96 (0.93-1.00) |  |
|  | Tier restrictions | 6.25 (6.08-6.42) | 0.94 (0.91-0.98) |  |
|  | Lockdown 2020-2021 | 6.33 (6.10-6.55) | 0.95 (0.91-1.00) |  |

Table S6. Rate of premature birth per 100 livebirths (95%CI), in each ethnicity group per deprivation quintile, in complete case dataset.

|  | White | Mixed | South Asian | Black | Other |
| --- | --- | --- | --- | --- | --- |
| Least deprived 20% | 5.5% (5.4-5.6) | 5.8% (4.9-6.7) | 6.2% (5.8-6.7) | 6.0% (5.1-6.9) | 5.2% (4.6-5.8) |
| Less deprived 20-40% | 5.9% (5.8-6.0) | 6.9% (6.0-7.8) | 6.5% (6.1-7.0) | 6.3% (5.5-7.1) | 5.8% (5.2-6.4) |
| Median deprived 40-60% | 6.7% (6.6-6.9) | 6.4% (5.7-7.0) | 7.2% (6.9-7.4) | 7.0% (6.6-7.4) | 6.2% (5.7-6.6) |
| More deprived 60-80% | 6.7% (6.6-6.8) | 6.8% (6.2-7.4) | 7.2% (6.9-7.3) | 6.9% (6.5-7.3) | 6.1% (5.7-6.5) |
| Most deprived 80-100% | 7.2% (7.1-7.3) | 7.2% (6.6-7.8) | 7.1% (6.8-7.3) | 6.7% (6.3-7.0) | 6.2% (5.8-6.6) |

Table S7. Spontaneous premature birth rates and rate ratios per subgroup in complete case dataset (adjusted for: deprivation, ethnicity, age, year, parity and previous adverse birth outcomes, maternal comorbidities, COVID-19 infection, and lockdowns)

| **Final Model: Spontaneous Premature Birth** | | | |
| --- | --- | --- | --- |
| **Total livebirths:** 1,111,045 | | | |
| **Deprivation** | **Rate per 100 livebirths (95%CI)** | **Rate ratio (95%CI)** | **P value** |
| Least deprived 20% | 2.38 (2.30-2.45) | Ref | <0.001 |
| Less deprived 20-40% | 2.59 (2.51-2.66) | 1.09 (1.04-1.13) |  |
| Median deprived 40-60% | 2.97 (2.90-3.04) | 1.25 (1.20-1.30) |  |
| More deprived 60-80% | 2.99 (2.92-3.05) | 1.26 (1.21-1.30) |  |
| Most deprived 80-100% | 3.15 (3.09-3.22) | 1.33 (1.28-1.38) |  |
| **Ethnicity** |  |  | <0.001 |
| White | 2.92 (2.88-2.95) | Ref |  |
| Mixed | 2.89 (2.68-3.10) | 0.99 (0.92-1.07) |  |
| South Asian | 2.74 (2.66-2.83) | 0.94 (0.91-0.97) |  |
| Black | 2.51 (2.38-2.64) | 0.86 (0.81-0.91) |  |
| Other | 2.61 (2.47-2.75) | 0.90 (0.85-0.95) |  |
| **Age** |  |  | <0.001 |
| 20-34 | 2.83 (2.80-2.87) | Ref |  |
| 13-19 | 4.03 (3.82-4.25) | 1.42 (1.35-1.50) |  |
| 35-44 | 2.81 (2.74-2.87) | 0.99 (0.96-1.02) |  |
| 45-55 | 2.67 (2.01-3.33) | 0.94 (0.74-1.21) |  |
| **Year** |  |  | 0.8616 |
| 2018 | 2.84 (2.77-2.91) | Ref |  |
| 2019 | 2.87 (2.81-2.94) | 1.01 (0.98-1.04) |  |
| 2020 | 2.87 (2.79-2.95) | 1.01 (0.97-1.06) |  |
| 2021 | 2.85 (2.66-3.05) | 1.00 (0.93-1.09) |  |
| **Parity and previous cesarean section** |  |  | <0.001 |
| Primiparous | 2.07 (1.35-2.79) | Ref |  |
| Multiparous & vaginal delivery | 4.91 (0.71-9.11) | 2.37 (0.71-7.90) |  |
| Multiparous & previous cesarean section | 4.58 (0.66-8.51) | 2.22 (0.67-7.37) |  |
| **Parity and previous PTB** |  |  | <0.001 |
| Primiparous | 6.75 (-0.41-13.16) | Ref |  |
| Multiparous & full-term | 1.31 (0.92-1.70) | 0.19 (0.05-0.75) |  |
| Multiparous & previous preterm birth | 5.70 (4.01-7.39) | 0.84 (0.22-3.28) |  |
| **Parity and previous stillbirth** |  |  | <0.001 |
| Primiparous | 2.38 (0.73-4.03) | Ref |  |
| Multiparous & livebirths | 3.66 (-0.44-7.76) | 1.54 (0.25-9.41) |  |
| Multiparous & previous stillbirth | 2.92 (-0.36-6.19) | 1.23 (0.20-7.50) |  |
| **Maternal comorbidities** |  |  | <0.001 |
| No health conditions | 2.91 (2.87-2.95) | Ref |  |
| Conditions diagnosed | 2.75 (2.69-2.81) | 0.95 (0.92-0.97) |  |
| **COVID** |  |  | <0.001 |
| Negative covid test | 2.86 (2.83-2.89) | Ref |  |
| Positive covid test | 3.92 (3.38-4.45) | 1.37 (1.20-1.57) |  |
| **Lockdown period** |  |  | 0.0012 |
| Pre-lockdown | 2.93 (2.88-2.99) | Ref |  |
| Lockdown 2020 | 2.79 (2.66-2.92) | 0.95 (0.90-1.00) |  |
| Tier restrictions | 2.65 (2.54-2.76) | 0.90 (0.86-0.95) |  |
| Lockdown 2020-2021 | 2.71 (2.56-2.85) | 0.92 (0.87-0.98) |  |

Table S8. Iatrogenic Preterm birth rates and rate ratios in complete case dataset (adjusted for: deprivation, ethnicity, the interaction between deprivation and ethnicity, age, year, parity and previous adverse birth outcomes, maternal comorbidities, COVID-19 infection, and lockdowns)

| **Final Model: Iatrogenic Preterm Births** | | | |
| --- | --- | --- | --- |
| **Total livebirths:** 1,111,045 | | | |
| **Deprivation** | **Rate per 100 livebirths (95%CI)** | **Rate ratio (95%CI)** | **P>z** |
| Least deprived 20% | 2.03 (1.96-2.10) | ref | <0.001 |
| Less deprived 20-40% | 2.13 (2.06-2.20) | 1.05 (1.00-1.10) |  |
| Median deprived 40-60% | 2.48 (2.42-2.54) | 1.22 (1.17-1.27) |  |
| More deprived 60-80% | 2.38 (2.32-2.44) | 1.17 (1.13-1.22) |  |
| Most deprived 80-100% | 2.64 (2.58-2.70) | 1.30 (1.25-1.35) |  |
| **Ethnicity** |  |  | <0.001 |
| White | 3.92 (3.38-3.86) | ref |  |
| Mixed | 3.90 (3.65-4.15) | 1.03 (0.83-1.27) |  |
| South Asian | 3.95 (3.84-4.05) | 1.16 (1.05-1.28) |  |
| Black | 3.86 (3.67-4.04) | 1.09 (0.89-1.34) |  |
| Other | 3.31 (3.14-3.47) | 0.88 (0.74-1.04) |  |
| **Age** |  |  | <0.001 |
| 20-34 | 3.71 (3.67-3.75) | Ref |  |
| 13-19 | 5.34 (5.08-5.60) | 1.44 (1.37-1.51) |  |
| 35-44 | 3.90 (3.83-3.98) | 1.05 (1.03-1.08) |  |
| 45-55 | 6.99 (6.04-7.94) | 1.89 (1.65-2.16) |  |
| **Year** |  |  | <0.001 |
| 2018 | 4.17 (4.08-4.26) | Ref |  |
| 2019 | 3.75 (3.67-3.83) | 0.90 (0.88-0.92) |  |
| 2020 | 3.67 (3.57-3.76) | 0.88 (0.85-0.91) |  |
| 2021 | 3.34 (3.16-3.53) | 0.80 (0.75-0.86) |  |
| **Parity & previous cesarean section** |  |  | <0.001 |
| Primiparous | 2.86 (1.66-4.07) | Ref |  |
| Multiparous & vaginal delivery | 5.21 (1.15-9.28) | 1.82 (0.55-6.05) |  |
| Multiparous & previous cesarean section | 6.38 (1.40-11.36) | 2.23 (0.67-7.41) |  |
| **Parity and previous premature birth** |  |  | <0.001 |
| Primiparous | 9.68 (-1.02-20.38) | ref |  |
| Multiparous & full-term | 1.83 (1.35-2.31) | 0.19 (0.05-0.74) |  |
| Multiparous & previous preterm birth | 6.66 (4.91-8.42) | 0.69 (0.18-2.70) |  |
| **Parity and previous stillbirth** |  |  | <0.001 |
| Primiparous | 2.77 (1.11-4.43) | Ref |  |
| Multiparous & livebirths | 5.82 (-1.35-12.93) | 2.10 (0.34-12.97) |  |
| Multiparous & previous stillbirth | 9.05 (-2.00-20.10) | 3.26 (0.53-20.15) |  |
| **Maternal Comorbidities** |  |  | <0.001 |
| None | 3.06 (3.02-3.10) | Ref |  |
| Conditions diagnosed | 5.56 (5.48-5.64) | 1.82 (1.78-1.85) |  |
| **COVID** |  |  | <0.001 |
| Negative covid test | 3.79 (3.75-3.82) | Ref |  |
| Positive covid test | 7.38 (6.68-8.09) | 1.95 (1.77-2.15) |  |
| **Lockdown period** |  |  | 0.4776 |
| Pre-lockdown | 3.80 (3.74-3.86) | Ref |  |
| Lockdown 2020 | 3.79 (3.63-3.94) | 1.00 (0.95-1.05) |  |
| Tier restrictions | 3.74 (3.61-3.88) | 0.99 (0.94-1.03) |  |
| Lockdown 2020-2021 | 3.90 (3.72-4.08) | 1.03 (0.97-1.08) |  |

Table S9. Premature birth incidence rates and ratios in cohort with COVID-19 positive patients excluded in complete case dataset (adjusted for: deprivation, ethnicity, the interaction between deprivation and ethnicity, age, year, parity and previous adverse birth outcomes, maternal comorbidities, COVID-19 infection, and lockdowns)

| **Final model: COVID-19 positive patients excluded subset** | | | |
| --- | --- | --- | --- |
| **Totals livebirths**: 1105691 | | | |
| **Deprivation** | **Rate per 100 livebirths (95%CI)** | **Rate ratio (95%CI)** | **P value** |
| Least deprived 20% | 5.59 (5.48-5.71) | Ref | <0.001 |
| Less deprived 20-40% | 5.98 (5.87-6.10) | 1.07 (1.04-1.10) |  |
| Median deprived 40-60% | 6.76 (6.66-6.86) | 1.23 (1.19-1.26) |  |
| More deprived 60-80% | 6.73 (6.63-6.83) | 1.22 (1.19-1.25) |  |
| Most deprived 80-100% | 7.08 (6.98-7.18) | 1.31 (1.27-1.34) |  |
| **Ethnicity** |  |  | 0.0107 |
| White | 6.50 (6.45-6.55) | Ref |  |
| Mixed | 6.63 (6.31-6.96) | 1.05 (0.89-1.23) |  |
| South Asian | 6.90 (6.76-7.04) | 1.13 (1.05-1.22) |  |
| Black | 6.61 (6.39-6.85) | 1.09 (0.93-1.27) |  |
| Other | 5.92 (5.71-6.13) | 0.94 (0.83-1.06) |  |
| **Age** |  |  | <0.001 |
| 20-34 | 6.36 (6.31-6.41) | Ref |  |
| 13-19 | 8.78 (8.45-9.10) | 1.38 (1.33-1.43) |  |
| 35-44 | 6.71 (6.61-6.80) | 1.05 (1.04-1.07) |  |
| 45-55 | 10.11 (8.96-11.26) | 1.59 (1.42-1.78) |  |
| **Year** |  |  | <0.001 |
| 2018 | 7.34 (7.23-7.46) | Ref |  |
| 2019 | 6.26 (6.16-6.35) | 0.85 (0.84-0.87) |  |
| 2020 | 6.18 (6.06-6.30) | 0.84 (0.82-0.87) |  |
| 2021 | 5.90 (5.63-6.17) | 0.80 (0.76-0.85) |  |
| **Parity and previous cesarean section** |  |  | <0.001 |
| Primiparous | 3.55 (3.49-3.61) | Ref |  |
| Multiparous & vaginal delivery | 29.99 (26.91-33.07) | 8.45 (7.53-9.48) |  |
| Multiparous & previous cesarean section | 37.61 (33.82-41.11) | 10.60 (9.47-11.87) |  |
| **Parity and previous PTB** |  |  | <0.001 |
| Primiparous | 15.25 (3.87-26.71) | Ref |  |
| Multiparous & full-term | 3.10 (2.48-3.71) | 0.20 (0.08-0.53) |  |
| Multiparous & previous preterm birth | 12.68 (10.16-15.20) | 0.83 (0.32-2.15) |  |
| **Parity and previous stillbirth** |  |  | <0.001 |
| Primiparous | 11.32 (3.53-19.11) | Ref |  |
| Multiparous & livebirths | 4.61 (3.37-5.85) | 0.41 (0.16-1.06) |  |
| Multiparous & previous stillbirth | 5.56 (4.04-7.08) | 0.49 (0.19-1.28) |  |
| **Maternal Comorbidities** |  |  | <0.001 |
| None | 5.79 (5.74-5.84) | Ref |  |
| Conditions diagnosed | 8.21 (8.11-8.30) | 1.42 (1.40-1.44) |  |
| **Lockdown period** |  |  | 0.0075 |
| Pre-lockdown | 6.60 (6.53-6.68) | Ref |  |
| Lockdown 2020 | 6.35 (6.15-6.55) | 0.96 (0.93-1.00) |  |
| Tier restrictions | 6.22 (6.05-6.39) | 0.94 (0.91-0.97) |  |
| Lockdown 2020-2021 | 6.34 (6.11-6.57) | 0.96 (0.92-1.00) |  |
